# Pathological Characterisation of Posterior Cortical Atrophy in Comparison With Amnestic Alzheimer's Disease

**DOI:** 10.1111/nan.70007

**Published:** 2025-04-02

**Authors:** Z. Abdi, K. X. Yong, J. M. Schott, A. Gatt, T. Revesz, S. J. Crutch, T. Lashley

**Affiliations:** ^1^ Department of Neurodegenerative Disease UCL Queen Square Institute of Neurology, University College London London UK; ^2^ The Queen Square Brain Bank for Neurological Disorders UCL Queen Square Institute of Neurology University College London London UK; ^3^ Dementia Research Centre, Department of Neurodegenerative Disease, UCL Queen Square Institute of Neurology University College London London UK; ^4^ UK Dementia Research Institute University College London London, UK

**Keywords:** Alzheimer's disease, atypical Alzheimer's disease, early‐onset Alzheimer's disease, posterior cortical atrophy, typical Alzheimer's disease, visual variant Alzheimer's disease

## Abstract

**Aims:**

Posterior cortical atrophy (PCA) is a predominantly young‐onset neurodegenerative syndrome, typically caused by Alzheimer's disease (PCA‐ad). PCA‐ad presents with visual and spatial dysfunction attributed to occipito‐parietal or ‘posterior’ brain regions rather than memory difficulties characteristic of typical amnestic‐led Alzheimer's disease (a‐ad) attributed to medial temporal regions. Imaging and neuropathological studies suggest that PCA‐ad is associated with a more posterior distribution of tau neurofibrillary tangles (NFTs), whereas β‐amyloid pathology (Aβ) is diffusely deposited throughout the cortex. This study characterised the neuropathological substrates of PCA‐ad in comparison with a‐ad, to further understanding of the biological basis of phenotypical heterogeneity in ad.

**Methods:**

Immunohistochemistry for Aβ; tau; the microglial markers CD68, CR3‐43 and Iba1; α‐synuclein; and TDP‐43 was carried out on 26 PCA‐ad and 27 age and gender‐matched a‐ad cases at the Queen Square Brain Bank. Aβ, tau and the three microglial markers were quantified in the superior frontal, superior temporal, superior parietal and occipital (primary visual cortex) cortices, with α‐synuclein and TDP‐43 assessed using formal staging criteria. In addition, microglial circularity, a morphological indicator of microglial activation state, was calculated.

**Results:**

There was a higher load of Aβ and tau in the parietal region of PCA‐ad compared to a‐ad. In the PCA‐ad compared to the a‐ad group, there were significant increases in tau load in parietal and frontal relative to temporal regions. There was no difference in cerebral amyloid angiopathy (CAA) severity between PCA‐ad and a‐ad. There was a significantly lower temporal CD68 load in a‐ad compared with PCA‐ad. In a‐ad, CD68 load was lowest and tau load highest in the temporal relative to all other regions.

**Conclusions:**

This study demonstrates differences in the distribution of Aβ and tau and variations in regional neuroinflammatory response in PCA‐ad and a‐ad. These findings extend our understanding of the biological substrates underpinning PCA‐ad and highlight the potential for exploring phenotypic variants to understand selective vulnerability in neurodegenerative diseases.

## Graphical abstract

There was a higher load of Aβ and tau in the parietal region of posterior cortical atrophy (PCA‐AD) compared to amnestic AD (a‐AD). In the PCA‐AD compared to the a‐AD group, there were significant increases in tau load in parietal and frontal relative to temporal regions. There was a significantly lower temporal CD68 load in a‐AD compared with PCA‐AD. This study demonstrates differences in the distribution of Aβ and tau, and variations in regional neuroinflammatory response in PCA‐AD and a‐AD.

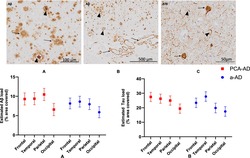

**Summary**

- There was a higher load of Aβ and tau in the parietal region of PCA‐ad compared to a‐ad.
- There was a significantly lower temporal CD68 load in a‐ad compared with PCA‐ad.
- There was a higher incidence of co‐occurring α‐syn in PCA‐ad compared to a‐ad.
- There were modest decreases in TDP‐43 in PCA‐ad compared with a‐ad.

**Abbreviations**
a‐ad
: amnestic ad
Aβ: amyloid beta
ad
: Alzheimer's disease
*ApoE*
: apolipoprotein E
AT8: Antiphospho‐tau antibody
CAA: cerebral amyloid angiopathy
CD68: cluster of differentiation 68
CR3–43: antimajor histocompatibility complex II, HLA‐DP, DQ, DR antibody
DAB: diaminobenzidine
DNA: deoxyribonucleic acid
EOAD: early‐onset Alzheimer's disease
HLA: Class II histocompatibility antigen
Iba1: ionised calcium‐binding adaptor molecule 1
IHC: Immunohistochemistry
MHC‐II: major histocompatibility Complex II
NFTs: neurofibrillary tangles
PCA: POSTERIOR cortical atrophy
PCR: polymerase chain reaction
p‐tau: phosphorylated tau
SD: standard deviation
TBST: tris‐buffered saline
TDP‐43: transactive response DNA binding protein
α‐Syn: alpha‐synuclein

## 1 Introduction

Posterior cortical atrophy (PCA) is often considered the most common atypical Alzheimer's disease (ad) clinical phenotype [1] and is typified by striking early deficits in higher order visuospatial processing, numeracy and literacy. These deficits are attributed to atrophy in the occipito‐parietal or ‘posterior’ brain regions with relatively spared memory, insight, language and capacity [2]. This is in contrast to typical amnestic‐led ad (a‐ad), which presents with memory problems from the outset, linked to atrophy in the medial temporal regions [3, 4]. PCA typically presents at a younger age compared to a‐ad, with the majority of patients aged 50–65 years [5, 6].

The ‘core’ ad pathologies, namely, Aβ plaques and tau neurofibrillary tangles (NFTs), are the most common underlying cause of PCA (PCA‐ad) reported in case series (62%–100%) [7, 8, 9]. A recent international study with access to postmortem data for 145 PCA cases found ad to be the primary neuropathological diagnosis in 94% [6] of cases. Lewy body disease, corticobasal degeneration [8, 9], transactive response DNA‐binding protein (TDP‐43) [6] non‐ad 3‐repeat tauopathy (Picks disease) [6] and very rarely prion disease [9, 10] have also been implicated as the primary underlying cause of PCA.

There has been an increasing interest in the correspondence between ‘biological subtypes’ of ad and clinical heterogeneity across the ad spectrum. Murray et al. [11] proposed the notion of the limbic predominant, hippocampal sparing and typical ad subtypes, based on the distribution of NFTs in postmortem brains. Atypical ad clinical phenotypes comprise a greater proportion of hippocampal sparing compared to typical and limbic predominant pathological subtypes, providing a means of exploring factors associated with regional vulnerability and sparing. Although the distribution of Aβ pathology is generally considered not to differ between PCA‐ad and a‐ad, imaging [12, 13, 14, 15] and neuropathological studies suggest that PCA‐ad is associated with a more posterior distribution of NFTs [7, 8, 16].

In PCA‐ad, investigations of regional vulnerability and pathological sparing have been essentially restricted to a small number of case reports and case series. There has been limited consideration of regional pathology and non‐ad copathology in PCA‐ad in relation to a‐ad. In line with increasing evidence suggesting that dysregulation of neuroinflammatory mechanisms is a core pathological feature of ad [17, 18], an altered inflammatory response is a candidate factor differentiating atypical ad including PCA‐ad from a‐AD [1]. Compared to a‐ad, disproportionate glial activation has been noted in the parietal relative to temporal regions in atypical ad [19], and genes implicated in microglial proliferation and phagocytosis (INPP5D) may have a comparable or reduced risk for PCA‐ad [20]. However, to our knowledge, there have been no pathological studies of neuroinflammatory changes in PCA‐ad.

This study aimed to understand the basis of extreme clinical heterogeneity in ad through a detailed neuropathological comparison of PCA‐ad and a‐ad. We compared regional Aβ, tau, cerebral amyloid angiopathy (CAA), α‐synuclein (α‐syn) and TDP‐43 burden across superior frontal, superior temporal, superior parietal and occipital (primary visual) regions. We also examined markers of activated microglia; cluster of differentiation 68 (CD68); the three isotopes of the major histocompatibility complex II, HLA‐DR, HLA‐DP and HLA‐DQ (detected using the CR3‐43 antibody); and ionised calcium‐binding adaptor molecule (Iba), a marker of all microglia irrespective of their activation state [21, 22].

We hypothesised differences in the regional distribution of ad pathology based on previous neuropathological and imaging studies. In particular, we expected the highest burden of tau pathology in the occipital and parietal regions both within the PCA‐ad group and in comparisons between the PCA‐ad and a‐ad groups. We hypothesised regional increases in microglial activation patterns in posterior cortical regions in PCA‐ad relative to a‐ad based on increased posterior ^11^C‐PBR28 binding in PCA‐ad and microglial markers in atypical ad [19, 23]. In PCA‐ad relative to a‐ad, we expected regional differences for ad pathology and neuroinflammation rather than copathology given previous pathological studies reporting a low burden of co‐pathology in atypical ad [24, 25].

## 2 Method

### 2.1 Case Selection

All selected cases were donated to the Queen Square Brain Bank for Neurological Disorders, UCL Queen Square Institute of Neurology. Twenty‐six PCA‐ad cases and 27 age and gender‐matched a‐ad cases were included in the study. All of the PCA‐AD cases fulfilled the consensus criteria for PCA‐AD [2]. Twenty‐five PCA‐AD cases fulfilled the criteria for PCA‐pure; one was classified as PCA‐plus, but the predominant underlying pathology was AD. All cases fulfilled the National Institute on Ageing‐Alzheimer's Association 2012 pathological guidelines for AD [26] and were end‐stage for AD neuropathology: Braak stage VI [27] for NFTs and Thal phase 5 [28] for Aβ. Summary demographic information for the PCA‐AD and a‐AD cases is shown in Table 1 (full details in Table S1).

**TABLE 1:** Summary demographic information for PCA‐AD and a‐AD.

Case	Gender	Age of onset (years)	Age at death (years)	Disease duration (years)	Postmortem delay (hours)	Brain weight (g)	Braak stage	Thal phase	*ApoE* status
Summary demographics									
PCA‐AD	12F 14M	57.5 (6.9)	67.6 (6.9)	10.1 (3.5)	63.4 (22)	1138 (119)	All VI	All 5	3/3 = 9 4/4 = 4 3/4 = 11 2/3 = 1 At least 1 *ApoE4* allele:15
a‐AD	14F 13M	59.5 (7.4)	72.3 (7.8)	12.8 (3.5)	65 (26)	1100 (172)	All VI	All 5	3/3 = 11 4/4 = 5 3/4 = 9 2/3 = 1 2/4 = 1 At least 1 *ApoE4* allele: 15
PCA‐AD vs a‐AD	*p* = 0.786	*p* = 0.319	*p* = 0.024*	*p* = 0.006*	*p* = 0.810	*p* = 0.357			*p* = 0.785

Abbreviation: SD, standard deviation.

^a^ *ApoE* genotyping for PCA‐AD case 20 was inconclusive, brain weight for a‐AD case 2 was not available,

* Significant at *p* < 0.05, mean (SD).

### 2.2 Immunohistochemistry (IHC)

IHC against Aβ, phosphorylated tau (p‐tau) for NFTs and neuropil threads, α‐syn, TDP‐43 and the microglial markers Iba1, CD68 and CR3‐43 was carried out manually. Sections were cut, mounted and stained with relevant antibodies (Table S2).

For the ad pathology and microglial markers, formalin‐fixed paraffin‐embedded blocks from the left superior frontal, superior temporal, superior parietal and occipital regions for both PCA‐ad and a‐AD were used to cut 8‐μm‐thick sections. Sections were deparaffinised in xylene and rehydrated using graded alcohols. Pretreatment for antigen retrieval was carried out using a pressure cooker in citrate buffer pH 6, aside from Aβ and α‐syn stained sections where slides were first pretreated using 98% formic acid for 10 min. Endogenous peroxidase activity was blocked using 0.3% hydrogen peroxide in methanol for 10 min. Nonspecific binding was blocked with 10% nonfat milk solution in tris‐buffered saline tween‐20 (TBS‐T, Thermofisher). Sections were incubated with primary antibodies for 1 h, followed by secondary antibodies and then Avidin‐Biotin complex (Vector) for 30 min with TBST washes in between. Activated diaminobenzidine (DAB) was used as a chromogen, and sections were counterstained with Mayer's Haematoxylin. The commonly accepted functions of the microglial proteins against which the markers used in this study are active are outlined in Table S3.

### 2.3 Apolipoprotein E (*ApoE*) Genotyping

Chipped frozen cerebellar tissue (≈ 100 mg) was homogenised in extraction buffer (0.1‐M sodium chloride, 20‐mM Trizma base, 25‐mM Ethylenediaminetetraacetic acid disodium and 0.5% SDS) and proteinase‐K solution (10 mg/mL) and allowed to digest at 55°C overnight. The homogenate was mixed with phenol, chloroform and isoamyl alcohol in a 1:1:1 ratio and centrifuged at 12,000 rpm. Subsequently, the aqueous layer was removed, 3‐M sodium acetate pH 5.3 added and deoxyribonucleic acid (DNA) precipitated with the addition of 100% ethanol. The precipitated DNA pellets were dried at room temperature, suspended in Tris‐EDTA and stored at 4°C.

Genotyping was carried out using the Qiagen polymerase chain reaction (PCR) Mix‐GC Rich kit with previously described primers [29]. PCR was run on the combined master mix and DNA solution at 94°C for 5 min, (94°C for 30 s, 60°C for 30 s and 72°C for 30 s) for 30 cycles, 72°C for 5 min and left at 4°C. Agarose (3% metaphor agarose gel/2% normal agarose) gel electrophoresis using GelRed dye was run for 1 h 30 min at 80 V and visualised in a DNR Bio‐Imaging Systems MiniBIS Pro for each sample.

### 2.4 Digital Image Analysis

All slides were digitised using an Olympus scanner at 20X magnification, and files were uploaded on the Olympus image analysis software. Using this software, an anatomically comparative cortical region of interest (all six cortical layers) within the superior frontal, superior temporal, superior parietal and primary visual cortex within the occipital lobe of each slide was extracted as a Tagged Image Format file. The selected regions are consistent with previous pathological studies on PCA‐ad [7, 8, 16, 30] and atypical ad [11, 19]. The sulcal margin of the superior frontal, superior temporal and superior parietal lobule were used for sampling. The superior (cuneus) or inferior (lingual) gyrus was used to sample the primary visual cortex, as it was impossible to differentiate between these two gyri on the relevant paraffin‐embedded block. The image processing software FIJI (https://imagej.net/software/fiji/) was used to quantify the percentage (%) surface area of DAB staining per section within each cortical region and used as the outcome measure of load for Aβ, tau pathology and the microglial markers [31].

CAA, α‐syn and TDP‐43 pathology were assessed according to commonly used staging criteria. CAA was assessed in all four cortical regions of the Aβ‐stained sections used for Aβ quantification as per the consensus protocol for the assessment of CAA in postmortem brain tissue [32] with scores of 0–3 assigned to parenchymal and meningeal CAA and capillary CAA assessed as present or absent. Neuropathological assessment of α‐syn pathology was carried out as per the commonly used Braak [33] and McKeith criteria [34, 35] and TDP‐43 using the Josephs criteria [36].

A measure of microglial circularity was calculated for the Iba1‐stained sections from the frontal, temporal, parietal and occipital cortices for the PCA‐ad and a‐ad cases and in the same region as the microglial marker quantification. Cell circularity was calculated as $\frac{4\mathrm{\pi CA}}{{\mathrm{CP}}^{2}}$, where CA = cell area and CP = cell perimeter. For cell circularity, the value of a perfect circle is 1, and the closer it is to 0, the more ramified the cell surface area. The Extended Particle Analyser plugin for FIJI was used to calculate circularity with options set to exclude any shape below a Feret diameter of 50. Feret is the distance between the furthest two points of an object, bounded by the smallest convex shape (convex hull) that contains that object. This filtering process is intended to ensure that as far as possible, the circularity measurement is applied to intact microglia with analysis of microglial fragments from different planes minimised. There were a small number (*N* = 8) of regions where zero (*N* = 2) or only one (*N* = 6) microglia ≥ 50 Ferets remained after the filtering process. These regions were excluded from the statistical analysis as a circularity measure based on ≤ 1 microglia was not felt to be representative of the microglial activity in that region.

### 2.5 Statistical Analysis

Data analysis was carried out using STATA, Version 17, and SPSS, Version 26. Graphs were drawn using GraphPad Prism Version 9.5.1. Differences between PCA‐ad and a‐ad demographics were calculated using an independent *t* test for normally distributed data and a Mann–Whitney *U* test for nonnormally distributed data. Gender frequency, *ApoE4* carrier status and non‐ad copathology as per Braak [33] and McKeith [37] neuropathological staging criteria for α‐syn and Jospeh's criteria for neuropathological staging of TDP‐43 [36] were compared between PCA‐ad and a‐ad using Fisher's exact test.

Pathology loads (% surface area) for Aβ and tau and microglial markers (Iba1, CD68 and CR3‐43) and circularity were analysed using mixed‐effects linear regression models including fixed effects for group and region and random effects for participant, allowing for different variances in participant groups. Interaction between fixed effects of group and region were included only if there was a priori justification (regional Aβ and tau load differing between PCA‐ad and a‐ad) [7, 8, 16] and/or they improved model fit (CD68).

As microglial circularity measures (median circularity) were skewed, a log transformation was used to improve the extent to which normality assumptions were satisfied. Comparisons between groups were subsequently expressed as percentage differences in geometric medians after back transformation. Pearson's correlation was used to correlate global CD68 and tau (averaged across all regions).

CAA severity ordinal measures (0 = absent, 1 = scant Aβ deposition, 2 = some circumferential Aβ deposition and 3 = widespread Aβ deposition) were analysed using a mixed‐effects ordered logistic regression model including fixed effects for group and region and random effects for participant. Capillary CAA (0 = absent and 1 = present) was analysed using a logistic regression model.

## 3 Results

### 3.1 Demographics

There were no significant differences between PCA‐ad and a‐ad in sex, age at disease onset, brain weight, time to postmortem and *ApoE4* carrier status (Table 1). The mean age at death for PCA‐ad patients was significantly lower at 67.7 (SD = 6.9) relative to a‐ad patients at 72.3 (SD = 7.8), *p* = 0.024. Accordingly, there was a significantly lower disease duration for the PCA‐ad patients of 10.1 (SD = 3.5) years versus 12.8 (SD = 3.5) for a‐ad patients, *p* = 0.006.

### 3.2 ad Pathology Load

**FIGURE 1:**
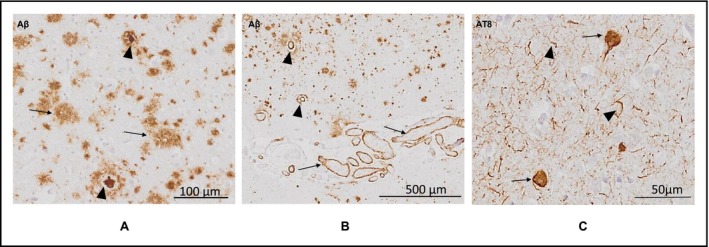
Microscopic pathological observations. (A) Arrow: diffuse Aβ plaques; arrowheads: dense core Aβ plaques. (B) Arrows: meningeal CAA; arrowheads: parenchymal CAA. (C) Arrows: NFTs; arrowheads: neuropil threads. Images were taken from the following cases (Table S1): A and B, PCA‐AD case 3; C, PCA‐AD case 6. Abbreviations: CAA, cerebral amyloid angiopathy; NFT, neurofibrillary tangles.

Microscopic pathological examination of Aβ and tau pathology showed similar findings in the PCA‐ad and a‐ad groups. IHC against Aβ revealed diffuse and dense core plaques, throughout the cortical layers in all regions examined (Figure 1A). Aβ deposition in cortical and meningeal arterial walls was observed as CAA (Figure 1B). IHC against p‐tau showed intraneuronal NFTs and neuropil threads (Figure 1C) throughout all regions examined, aside from the line of Gennari in the occipital region, which is classically reported to be devoid of significant tau immunoreactivity [38].

**FIGURE 2:**
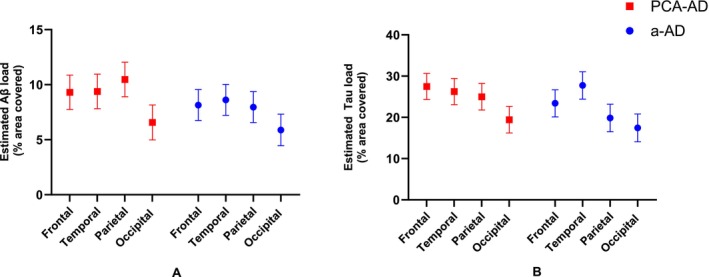
Estimated (A) Aβ and (B) tau % surface area by region in the PCA‐AD and a‐AD groups. Error bars correspond to 95% CIs. In the PCA‐AD compared to the a‐AD group, tests of differences between interaction terms provided evidence of increases in tau load in parietal and frontal relative to temporal regions (vs. parietal: *p* = 0.014; vs. frontal; *p* = 0.035).

The estimated % Aβ and tau surface area for each region in the PCA‐ad and a‐ad groups are detailed in Figure 2. Averaged across regions, there was no evidence that overall Aβ or tau load differed between PCA‐ad and a‐ad groups (*p* = 0.146, *p* = 0.156, respectively). However, at the regional level, there was evidence of higher parietal Aβ and tau load in the PCA‐ad compared to the a‐ad group, being increased by 2.5% (95%CI [0.4, 4.7]) and 5.2% (95%CI [0.5, 9.9]) in surface area, respectively (Figure 2; Table S4). At the individual level, the PCA‐ad case with PCA‐plus clinical features had average parietal tau load despite having the highest parietal Aβ load within the PCA group. See the Supporting Information for results including and excluding this case.

Formal tests of differences between interaction terms provided evidence that the effects of region on tau distribution differed between groups. In the PCA‐ad compared to the a‐ad group, there was evidence of increases in tau load in parietal and frontal relative to temporal regions (vs. parietal: *p* = 0.014; vs. frontal; *p* = 0.035; Figure 2B). There was no evidence that the effect of region on Aβ load differed between PCA‐ad and a‐ad groups.

### 3.3 Cerebral Amyloid Angiopathy

**FIGURE 3:**
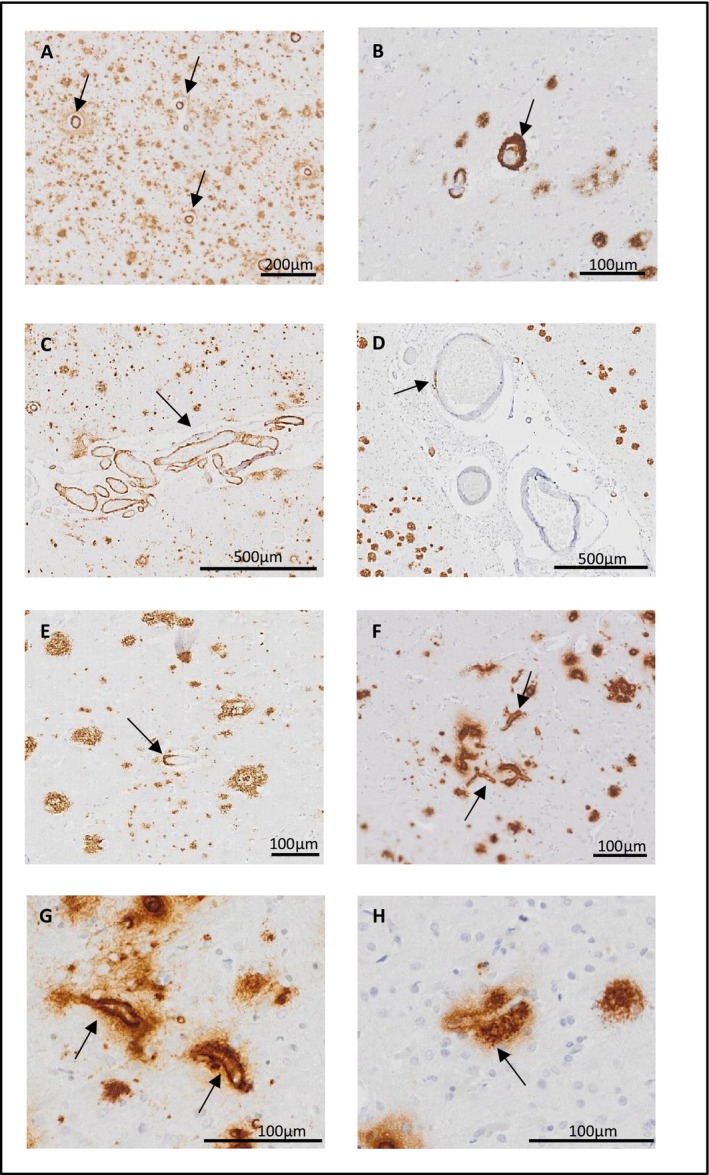
Representative images of CAA pathology and associated scoring of Aβ‐stained sections. (A) parenchymal CAA (score 3 = widespread circumferential Aβ). (B) double barrel vessel with circumferential Aβ. (C) meningeal CAA (score 3 = widespread circumferential Aβ). (D) meningeal CAA (score 1 = scant Aβ deposition). (E) parenchymal CAA (score 1 = scant Aβ deposition). (F, G) Capillary CAA. (H) capillary with perivascular Aβ deposition. Images were taken from the following cases (Table S1): A and C, PCA‐AD Case 3; B, a‐AD Case 10; D, PCA‐AD Case 13; E, a‐AD Case 4; F, a‐AD Case 1; G, PCA‐AD Case 17; H, PCA‐AD Case 23. Abbreviations: CAA, cerebral amyloid angiopathy.

The neuropathological features of CAA were similar in PCA‐ad and a‐ad. The majority of cases in both PCA‐ad and a‐ad had circumferential or patchy Aβ deposits as CAA in parenchymal (Figure 3A,B,E) and meningeal (Figure 3C,D) arteries and arterioles in all four examined cortices.

Both PCA‐ad and a‐ad cases were almost devoid of capillary CAA in the frontal, temporal and parietal cortices with the highest incidence of capillary CAA observed in the occipital cortex for both groups (Figure 3F,G). In the occipital cortex, capillaries, either with or without Aβ deposits in their walls, often showed perivascular Aβ deposits (Figure 3H).

**FIGURE 4:**
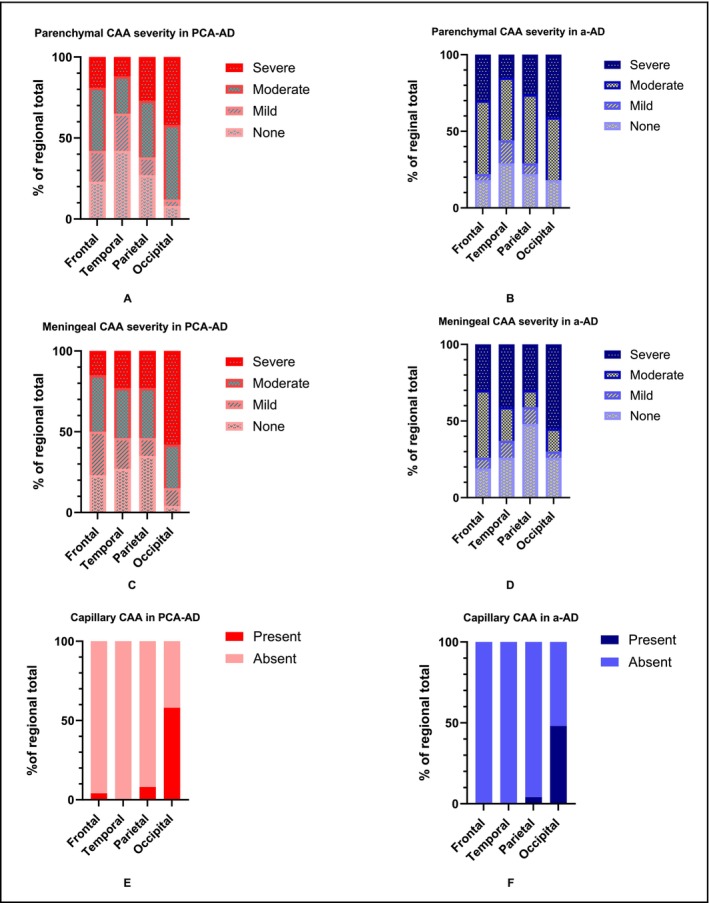
Observed CAA severity in PCA‐AD and a‐AD. Parenchymal (A, B), meningeal (C, D) and capillary vessels (E, F). Abbreviations: CAA, cerebral amyloid angiopathy.

There was no evidence that overall parenchymal or meningeal CAA load averaged across all regions differed between the PCA‐ad and a‐ad groups (parenchymal: *p* = 0.409; meningeal: *p* = 0.682). Observed CAA was consistent with greater severity of both parenchymal and meningeal CAA and frequency of capillary CAA in the occipital relative to all other regions (Figure 4).

### 3.4 Microglial Load

**FIGURE 5:**
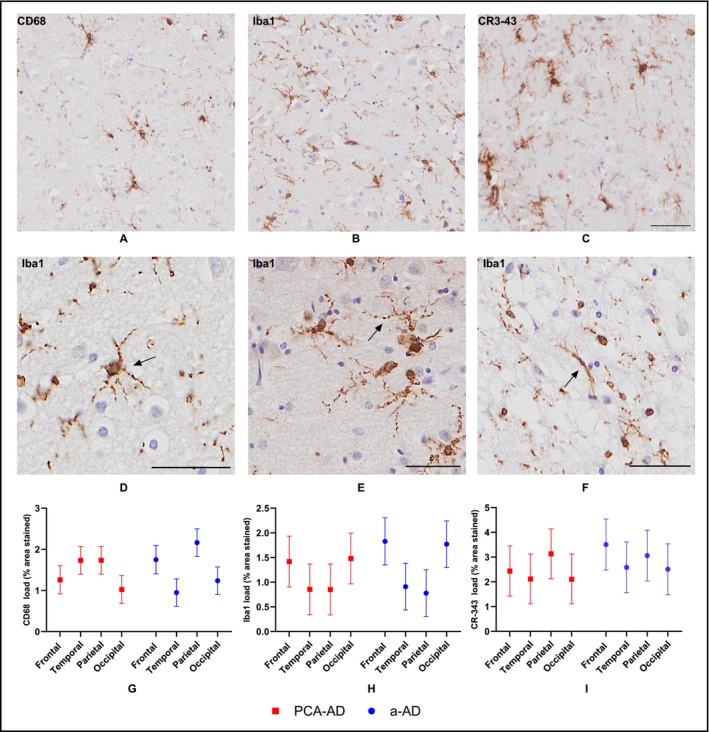
Representative images of microglial staining and results of microglial load. (A–C) Sections stained for CD68, Iba1 and CR3‐43 in the frontal cortex of the same PCA‐AD case. The scale bars represent 100 μm. (D, E) Iba1‐stained dystrophic microglia with beaded processes. (F) An example of a rod‐shaped microglia, a marker of dystrophy. The scale bars represent 50 μm in images. (G) Estimated regional CD68 % surface area of PCA‐AD and a‐AD groups. (H) Estimated regional Iba1 % surface area of PCA‐AD and a‐AD groups. (I) Estimated regional CR3‐43 % surface area of PCA‐AD and a‐AD groups. Error bars correspond to 95% CIs. In the PCA‐AD compared to the a‐AD group, tests of differences between interaction terms provided evidence of increased CD68 load in temporal relative to all other regions (all *p* < 0.001). Images were taken from the following cases (Table S1): A–C, PCA‐AD Case 7; D, a‐AD Case 11; E, a‐AD Case 20; F, a‐AD Case 15.

Representative images of CD68, Iba1, and CR3‐43 IHC in the frontal cortex are shown in Figure 5A–C. CD68 staining was predominantly confined to the microglial cell body, whereas Iba1 and CR3‐43 staining highlighted both the microglial cell body and processes. There was evidence of microglial dystrophy on Iba1‐stained sections with beading of the microglial processes and evidence of rod‐shaped microglia, a marker of dystrophy (Figure 5D–F).

Estimated CD68, Iba1 and CR3‐43 % surface area for each region in the PCA‐ad and a‐ad groups are detailed in Figure 5G–I. Averaged across regions, there was no evidence that overall CD68 load differed between PCA‐ad and a‐ad groups (*p* = 0.548). However, at the regional level, there was evidence of higher temporal CD68 load and lower frontal CD68 load in the PCA‐ad compared to the a‐ad group. In PCA‐ad relative to the a‐ad group, CD68 surface area was increased by 0.8% (95%CI [0.3, 1.3]) in the temporal region and decreased by 0.5% (95%CI [0.02, 1.0]) in the frontal region (Table S4).

Formal tests of differences between interaction terms provided evidence that the effect of region on CD68 load differed between PCA‐ad and a‐ad groups. In the PCA‐ad compared to the a‐ad group, there was evidence of increased CD68 load in temporal relative to all other regions (all *p* < 0.001), driven by the low temporal CD68 load in the a‐ad group (Figure 5G). There was no evidence of group differences in Iba1 or CR3‐43 loads averaged across regions (Figure 5H,I) or evidence that the effect of region on Iba1 or CR3‐43 load differed between groups.

### 3.5 Microglial Circularity

**FIGURE 6:**
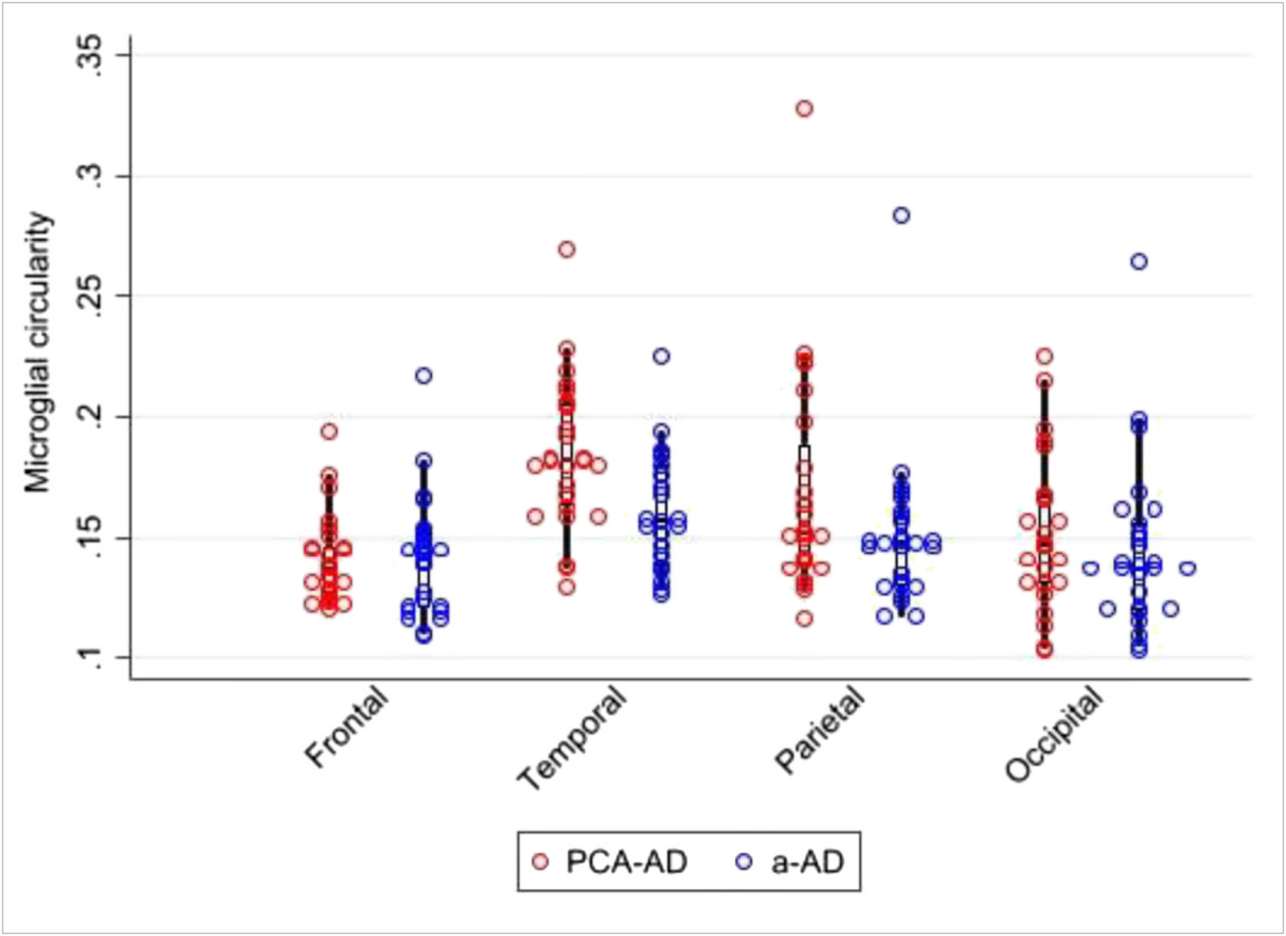
Bubble plot illustrating microglial circularity results for PCA‐AD and a‐AD. Increasing circularity indicates a more amoeboid, less branched shape, seen in more activated microglia.

Observed median microglial circularity, a morphological marker of activation in Iba1 positive microglia, suggests a tendency towards higher circularity in the PCA‐ad relative to the a‐ad group (Figure 6). Averaged across regions, there was evidence that microglial circularity was higher in PCA‐ad compared with a‐ad groups (estimated % increase in circularity: 8.7% (95% CI [1.4, 15.9])).

### 3.6 Relationship Between CD68 and Tau

We observed a seemingly inverse relationship between CD68 and tau. Notably, in a‐ad, CD68 load was lowest and tau load highest in the temporal relative to all other regions. In exploratory analyses, we therefore assessed the association between CD68 and tau across all regions and both groups. There was a positive association between global CD68 and global tau averaged across regions (*r* [51] = 0.42, *p* = 0.002; Figure S1A). However, despite this global positive association, in the a‐ad group, observed within‐participant ratios of tau to CD68 were highest in the temporal relative to all other regions, suggesting a tendency for a discrepancy in this relationship at the regional level (Figure S1B).

### 3.7 Non‐ad Copathology

**FIGURE 7:**
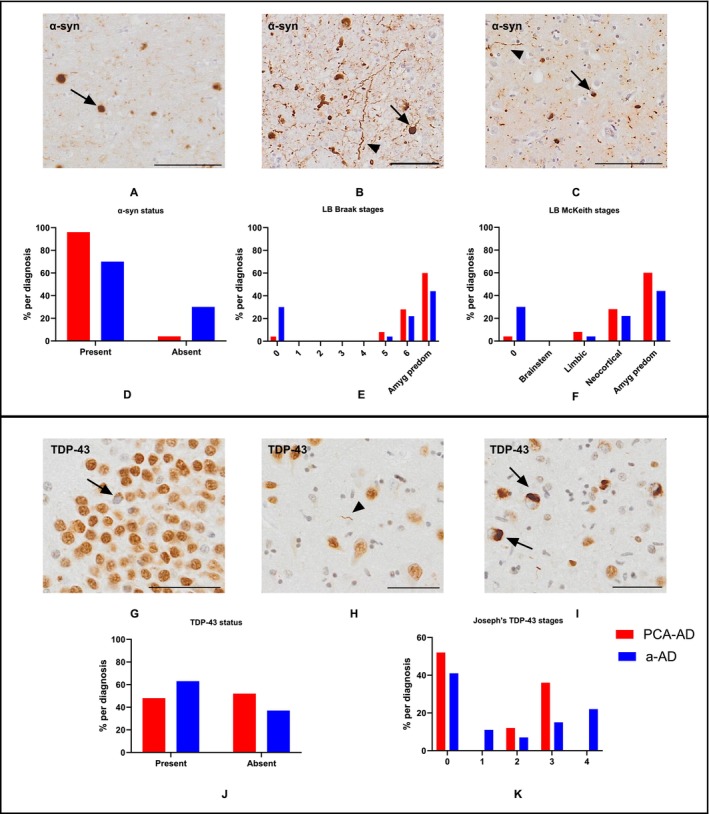
Representative images and results for α‐syn and TDP‐43. (Top panel) α‐Syn stained Lewy bodies (arrows) and Lewy neurites (arrowheads) in the (A) entorhinal cortex, (B) amygdala and (C) temporal cortex. α‐Syn frequency (% per diagnosis) in PCA‐AD and a‐AD using (D) dichotomised results into present/absent, the (E) Braak classification system and the (F) McKeith criteria. (Bottom panel) TDP‐43 stained cytoplasmic inclusions (arrows) and threads (arrowhead) in the (G) hippocampal dentate gyrus, (H) subiculum and (I) amygdala. TDP‐43 frequency (% per diagnosis), in PCA‐AD and a‐AD using (J) dichotomised results into present/absent, and the (K) Josephs 2014 criteria. Images were taken from the following cases (Table S1): A, a‐AD Case 14; B, PCA‐AD Case 6; C, a‐AD Case 14; G‐I PCA‐AD, Case 22. The scale bars represent 50 μm in images. Abbreviations: Amyg predom, Amygdala predominant; LB, Lewy body.

Representative images for α‐syn and TDP‐43 are shown in Figure 7A–C and Figure 7G–I, respectively. When α‐syn status was dichotomised into present/absent, the observed proportion of patients with α‐syn present was higher in PCA‐ad compared to a‐ad (Figure 7D). Amygdala predominant was the most prevalent category in both PCA‐ad and a‐ad groups as per both the Braak (Figure 7E) and McKeith (Figure 7F) staging criteria followed by Braak Stage 6 (equivalent to the McKeith neocortical category) for PCA‐ad (28%) and none for a‐ad (30%). However, there was no evidence of differences in the α‐syn stage (Braak: *p* = 0.09; McKeith: *p* = 0.130).

When the TDP‐43 status was dichotomised into present/absent, the observed proportion of patients with TDP‐43 present was comparable between groups (Figure 7J). The most common TDP‐43 category was ‘none’ in both groups. Stage 3 (spread to the dentate/fusiform gyrus) was the next most common category in PCA‐ad and Stage 4 (spread to inferior temporal gyrus) in a‐ad. There was evidence of reduced TDP‐43 severity as per the Josephs staging criteria in PCA‐ad compared to a‐ad groups (*p* = 0.024), driven by the observation that 22% (*N* = 6) of a‐ad cases had Stage 4 TDP‐43 pathology compared with none of the PCA‐ad group (Figure 7K).

There was no statistically significant association between *ApoE4* carrier status and pathological observations. A summary of results can be found in the Supporting Information.

## 4 Discussion

This study demonstrated a phenotype‐consistent distribution of ‘classical’ ad pathology with higher Aβ and tau in the parietal region in PCA‐ad compared to a‐ad. This finding is in keeping with previous neuropathological and molecular imaging studies showing an increased occipito‐parietal tau burden in PCA‐ad compared with a‐ad [7, 8, 12, 13, 14, 15, 16]. There was also a higher frontal tau load in PCA‐ad compared with a‐ad. Although this finding might seem counterintuitive for a ‘posterior’ predominant syndrome, it is supported by recent in vivo evidence for the frontal region as a particularly vulnerable region for accumulation of tau over time in PCA‐ad despite a posterior predominant distribution pattern in the occipito‐parietal regions earlier in the disease course [39]. This work therefore provides further evidence that clinical phenotypes of ad reflect underlying neuroanatomical variations in ad pathology and, more specifically, regional variations in tau burden.

Although the implications of this higher frontal tau burden for the PCA‐ad phenotype are not clear, the observation may reflect an anterior progression of tau due to an ‘oversaturation’ of posterior regions with tau pathology. Alternatively, this finding may reflect the spread of tau along networks connecting the posterior and anterior cortical regions. This interpretation is in keeping with previous findings of hypometabolism in the frontal eye field regions of PCA‐ad compared with a‐ad [40]. Although the authors of this study had suggested this finding could be due to loss of signal input from the occipito‐parietal region, the higher frontal tau burden suggests it may be due to the anterior progression of pathology.

On the whole, evidence from histological studies suggests higher levels of microglial markers including CD68 and CR3‐43 in ad compared with controls but results are more mixed for other markers such as Iba1 [22, 41]. Higher neuroinflammation in the parietal region of atypical ad cases compared with a‐ad cases at postmortem has previously been described [19]. A study of neuroinflammation in PCA‐ad used TSPO positron emission tomography, a marker of microglial activation, finding increased binding in the occipital region of PCA‐ad compared with a‐ad [23].

Our study demonstrated differences in regional neuroinflammatory patterns between PCA‐ad and a‐ad. CD68 load, a marker of phagocytic properties of microglia, was higher in superior temporal and lower in superior frontal regions in the PCA‐ad compared to the a‐ad group. Regional increases in tau and decreases in markers of activated microglia also differed between PCA‐ad and a‐ad, with an inverse relationship between tau and CD68 load in the temporal region of the a‐ad group. Furthermore, the microglia in the PCA‐ad group had overall higher circularity, especially in the temporal region, an indication of a more activated microglial phenotype compared with a‐ad. Microglia are the resident scavenger cells in the brain responsible for phagocytosis as part of the innate immune system. Taken together, higher temporal CD68 load and higher microglial circularity in PCA‐ad may indicate a more organised innate immune response; conversely, in the a‐ad group, there was evidence for a more dysfunctional, perhaps tau‐overloaded, microglial response in the temporal region. Although other interpretations are possible, these findings may be interpreted in terms of a disconnect between microglial phagocytic activity at later stages of ad leading to further accumulation of tau and subsequent neuronal death [42, 43, 44].

To our knowledge, this is the first study to investigate CAA in PCA‐ad compared with a‐ad. We did not find evidence that CAA severity differed between PCA‐ad and a‐ad, with the observed CAA severity pattern in both groups in keeping with the widely observed finding of numerous neuropathological studies, showing a predilection of CAA for the occipital region in ad [45, 46, 47]. This finding may be in keeping with the broader observations that Aβ appears less able to segregate ad variants based on regional deposition patterns due to a more diffuse deposition throughout the cortex [14, 48]. In the case of CAA, however, the primary driver in regional variation is the strong predilection of CAA for the occipital region beyond which the additional regional contribution of CAA to phenotypic variation is difficult to assess.

We investigated α‐syn and TDP‐43 frequency according to commonly used staging criteria. The findings for α‐syn were mixed and showed that although there was no difference in severity between PCA‐ad and a‐ad, α‐syn was almost ubiquitous in PCA‐ad, with 96% of cases having concurrent α‐syn pathology, compared with 74% of a‐ad cases. The relevance of this higher a‐syn incidence in PCA‐ad, despite this group having a shorter disease duration, is not clear. Notably, the amygdala predominant category was the most common category for α‐syn deposition in both PCA‐ad (60%) and a‐ad (44%). This either suggests that despite higher incidence, α‐syn does not play a significant role in the PCA‐ad phenotype, or, if it does, this is not a direct reflection of its anatomical deposition pattern and may modulate the disease phenotype in alternative ways.

This study is in keeping with the observation of synucleinopathy reported in a small number of PCA‐ad neuropathological case series [8, 9], although direct statistical comparisons with a‐ad are lacking. The incidence of α‐syn in almost all PCA‐ad cases in this study is perhaps surprising, and it must be noted that all but one of the PCA‐ad cases in the present study were classified as PCA‐pure with no clinical signs compatible with an alternative or concurrent neurodegenerative disease at the time of diagnosis, including synucleinopathy. Therefore, the relevance of the high incidence of α‐synuclein co‐pathology in these cases is unclear.

Despite a comparable incidence of TDP‐43 in the dichotomised (TDP‐43 absent/present) results, TDP‐43 severity was lower in PCA‐ad compared with age‐matched a‐ad; indeed, half (51%) of all PCA‐ad cases did not have any TDP‐43, suggesting that TDP‐43 likely does not play a major role in influencing the clinical phenotype of ad. These findings are in keeping with the literature, with atypical ad in general considered to have less comorbid pathology [49, 50, 51, 52, 53].

One potential limitation of the work presented here is that due to age matching; the age of onset of 59 years is relatively young for ad and would classify most a‐ad cases as having young‐onset ad, considered to be an age at onset < 65 years. Although having groups of comparable age would be considered important for a comparative neuropathological study, this may have implications for the generalisability of the results to a more ‘typical’ a‐ad group with older age at onset. Furthermore, there are several sources of variation in the use of IHC methods in human brain tissue, which may lead to unintended differences between studies. These include variations in tissue fixation including the duration of brain fixation in formalin, thickness of brain sections and variations in the numerous steps involved in IHC protocols. In addition, we do not have the cause of death for all subjects. There is a theoretical possibility that microglial activity in particular may be differentially affected by the cause of death, although we have no direct evidence from our work to suggest this.

To date, the biological mechanisms driving clinical heterogeneity in ad are largely unknown. Regional variations in ad pathology, differential patterns of neuroinflammation, differences in comorbid pathology and intrinsic local tissue factors underlying differences in selective vulnerability are all potential candidates for the biological basis of the heterogeneity observed across the clinical spectrum of ad. This study provides evidence for regional variations in ad pathology, neuroinflammatory patterns and to a lesser extent copathology in human postmortem brains of PCA‐ad compared with a‐ad cases, despite both groups having end‐stage ad neuropathologically. This is in line with longitudinal imaging studies showing that phenotypic variations in atrophy and tau pathology [54, 55, 56, 57] are maintained during the disease course and pathological studies at post‐mortem [7, 8, 11, 16, 58]. This study highlights the potential for in‐depth analyses of atypical AD phenotypes in furthering the understanding of the clinical heterogeneity of AD, and in due course identifying its causes, with the ultimate aim of tailored disease‐modifying treatment.

## Author Contributions

**Abdi Z:** conceptualisation, investigation, formal analysis, original draft preparation (lead). **Yong KX**: formal analysis, original draft preparation – review and editing. **Schott JM**: supervision, original draft preparation – review and editing. **Gatt A**: investigation. **Revesz T**: investigation. **Crutch SJ**: supervision, original draft preparation – review and editing. **Lashley T**: supervision, original draft preparation – review and editing.

## Ethics Statement

Ethical approval was granted, along with an approved MTA, for the use of the tissue in the project.

## Conflicts of Interest

The authors declare no conflicts of interest.

## Supporting information


**Table S1** Demographic information for PCA‐ad and a‐ad cases.
**Table S2** Details of antibodies used.
**Table S3** Microglial protein location of expression and function ^21,22,29,30^.
**Figure S1** Relationship between tau and CD68. (A) correlation between CD68 and tau showing positive association between CD68 and tau load globally (CD68 and tau load has been averaged across all four brain regions for PCA‐ad and a‐ad). (B) Tau to CD68 % surface area ratio in PCA‐ad and a‐ad cases.
**Table S4** Estimated group differences [95% CIs] in Aβ, tau and CD68 markers (%area) at each region. Positive values indicate higher load in PCA‐ad relative to a‐ad.

## Data Availability

The data that support the findings of this study are available on request from the corresponding author. The data are not publicly available due to privacy or ethical restrictions.
