# Supplementary material for: Pathological Characterisation of Posterior Cortical Atrophy in Comparison With Amnestic Alzheimer's Disease
Source: Neuropathol Appl Neurobiol. 2025 Apr 2;51(2):e70007. doi: 10.1111/nan.70007 (PMC11964714; doi:10.1111/nan.70007)
Supplement: Supplementary file 1 — Table S1 Demographic information for PCA‐ad and a‐ad cases. Table S2 Details of antibodies used. Table S3 Microglial protein location of expression and function 21,22,29,30. Figure S1 Relationship between tau and CD68. (A) correlation between CD68 and tau showing positive association between CD68 and tau load globally (CD68 and tau load has been averaged across all four brain regions for PCA‐ad and a‐ad). (B) Tau to CD68 % surface area ratio in PCA‐ad and a‐ad cases. Table S4 Estimated group differences [95% CIs] in Aβ, tau and CD68 markers (%area) at each region. Positive values indicate higher load in PCA‐ad relative to a‐ad. [file NAN-51-e70007-s001.docx]

**Table S1. Demographic information for PCA-AD and a-AD cases.**

| **Case** | **Gender** | **Age of**  **onset (yrs)** | **Age at**  **death (yrs)** | **Disease**  **Duration (yrs)** | **Post-mortem**  **Delay (hrs/mins)** | **Brain weight(g)** | **Braak and Braak** | **Thal phase** | **ApoE status** |
| --- | --- | --- | --- | --- | --- | --- | --- | --- | --- |
| **PCA-AD cases n=26** | | | | | | | | | |
| **1** | **M** | **56** | **62** | **6.0** | **70:55** | **1208** | **6** | **5** | **4/4** |
| **2** | **F** | **52** | **60** | **8.0** | **105:15** | **1268** | **6** | **5** | **3/3** |
| **3** | **M** | **57** | **69** | **12.0** | **59:50** | **1161** | **6** | **5** | **3/3** |
| **4** | **F** | **46** | **60** | **14.0** | **47:30** | **797** | **6** | **5** | **3/4** |
| **5** | **M** | **58** | **68** | **10.0** | **52:05** | **1154** | **6** | **5** | **3/4** |
| **6** | **M** | **55** | **65** | **10.0** | **96:30** | **1270** | **6** | **5** | **3/3** |
| **7** | **F** | **49** | **55** | **6.0** | **47:50** | **1100** | **6** | **5** | **3/3** |
| **8** | **F** | **55** | **68** | **13.0** | **86:50** | **1126** | **6** | **5** | **3/4** |
| **9** | **F** | **57** | **65** | **8.0** | **46:35** | **1121** | **6** | **5** | **3/4** |
| **10** | **M** | **68** | **76** | **8.0** | **50:20** | **1315** | **6** | **5** | **3/3** |
| **11** | **M** | **60** | **71** | **11.0** | **47:35** | **1018** | **6** | **5** | **3/3** |
| **12** | **F** | **64** | **74** | **10.0** | **55:15** | **1159** | **6** | **5** | **4/4** |
| **13** | **F** | **54** | **66** | **12.0** | **85:30** | **1119** | **6** | **5** | **3/4** |
| **14** | **M** | **63** | **75** | **12.0** | **93:28** | **1151** | **6** | **5** | **2/3** |
| **15** | **F** | **70** | **87** | **17.0** | **51:10** | **1050** | **6** | **5** | **3/4** |
| **16** | **F** | **52** | **64** | **12.0** | **70:15** | **944** | **6** | **5** | **3/4** |
| **17** | **F** | **62** | **68** | **6.0** | **77:30** | **1074** | **6** | **5** | **4/4** |
| **18** | **M** | **61** | **72** | **11.0** | **27:25** | **1024** | **6** | **5** | **3/4** |
| **19** | **F** | **58** | **62** | **4.0** | **92:20** | **1234** | **6** | **5** | **3/4** |
| **20** | **M** | **62** | **67** | **5.0** | **31:15** | **1210** | **6** | **5** | **non-conclusive** |
| **21** | **M** | **50** | **61** | **11.0** | **78:20** | **1144** | **6** | **5** | **3/4** |
| **22** | **M** | **65** | **72** | **7.0** | **58:13** | **1110** | **6** | **5** | **4/4** |
| **23** | **M** | **58** | **67** | **9.0** | **35:27** | **1223** | **6** | **5** | **3/3** |
| **24** | **M** | **44** | **59** | **15.0** | **90:45** | **1338** | **6** | **5** | **3/4** |
| **25** | **F** | **70** | **78** | **8.0** | **45:40** | **1042** | **6** | **5** | **3/3** |
| **26** | **M** | **50** | **67** | **17.0** | **44:25** | **1252** | **6** | **5** | **4/4** |

**cont next pag**

| **Case** | **Gender** | **Age of**  **onset (yrs)** | **Age at**  **death (yrs)** | **Disease**  **Duration (yrs)** | **Post-mortem**  **Delay (hrs/mins)** | **Brain weight(g)** | **Braak and Braak** | **Thal phase** | **ApoE status** |
| --- | --- | --- | --- | --- | --- | --- | --- | --- | --- |
| **a-AD cases n=27** | | | | | | | | | |
| **1** | **F** | **59** | **66.0** | **7.0** | **95:00** | **1100** | **6** | **5** | **4/4** |
| **2** | **M** | **64** | **71.0** | **7.0** | **124:15** | **Not available** | **6** | **5** | **3/4** |
| **3** | **M** | **57** | **64** | **7** | **28:30** | **1160** | **6** | **5** | **3/4** |
| **4** | **M** | **65** | **75** | **10** | **42:45** | **949** | **6** | **5** | **3/3** |
| **5** | **F** | **67** | **76** | **9** | **82:15** | **988** | **6** | **5** | **3/4** |
| **6** | **M** | **64** | **77** | **13** | **90:05** | **1264** | **6** | **5** | **4/4** |
| **7** | **F** | **62** | **76** | **14** | **26:45** | **1028** | **6** | **5** | **3/4** |
| **8** | **F** | **65** | **77** | **12** | **99:00** | **902** | **6** | **5** | **3/4** |
| **9** | **F** | **56** | **66** | **10** | **51:20** | **811** | **6** | **5** | **4/4** |
| **10** | **F** | **70** | **86** | **16** | **90:20** | **1065** | **6** | **5** | **3/4** |
| **11** | **M** | **69** | **81** | **12** | **78:15** | **1116** | **6** | **5** | **3/3** |
| **12** | **F** | **51** | **62** | **11** | **62:55** | **978** | **6** | **5** | **3/4** |
| **13** | **M** | **72** | **88** | **16** | **85:35** | **1120** | **6** | **5** | **3/4** |
| **14** | **M** | **55** | **67** | **12** | **28:35** | **1015** | **6** | **5** | **3/3** |
| **15** | **M** | **52** | **69** | **17** | **35:04** | **891** | **6** | **5** | **3/3** |
| **16** | **M** | **63** | **73** | **10** | **31:10** | **1269** | **6** | **5** | **3/3** |
| **17** | **F** | **49** | **62** | **13** | **76:40** | **996** | **6** | **5** | **3/3** |
| **18** | **M** | **52** | **68** | **16** | **73:45** | **1234** | **6** | **5** | **3/3** |
| **19** | **F** | **60** | **73** | **13** | **67:00** | **1128** | **6** | **5** | **2/4** |
| **20** | **M** | **56** | **65** | **9** | **90:30** | **1350** | **6** | **5** | **3/3** |
| **21** | **M** | **63** | **79** | **16** | **58:10** | **1084** | **6** | **5** | **2/3** |
| **22** | **F** | **57** | **76** | **19** | **61:19** | **1423** | **6** | **5** | **3/3** |
| **23** | **M** | **52** | **71** | **19** | **57:50** | **1303** | **6** | **5** | **3/4** |
| **24** | **M** | **54** | **67** | **13** | **45:35** | **1097** | **6** | **5** | **3/3** |
| **25** | **F** | **58** | **72** | **14** | **32:15** | **1458** | **6** | **5** | **4/4** |
| **26** | **F** | **43** | **58** | **15** | **81:26** | **820** | **6** | **5** | **4/4** |
| **27** | **M** | **63** | **79** | **16** | **58:50** | **1075** | **6** | **5** | **3/3** |

**Table S2**. Details of antibodies used.

| Primary antibody | Catalogue Number | Species | Dilution | Pre- treatments | Secondary antibody |
| --- | --- | --- | --- | --- | --- |
| Aβ | Dako  (M0872) | Mouse | 1:100 | FA+PC | Rabbit anti-mouse biotinylated |
| AT8 (against p-tau) | Thermo (MN1020) | Mouse | 1:600 | PC | Rabbit anti-mouse biotinylated |
| CD68 | Dako  (M0876) | Mouse | 1:100 | PC | Rabbit anti-mouse biotinylated |
| Iba1 | Wako  (019-19741) | Rabbit | 1:1000 | PC | Swine anti-rabbit  Biotinylated |
| CR3-43 | Dako  (M0775) | Mouse | 1:150 | PC | Rabbit anti-mouse biotinylated |
| α-Synuclein | Abcam (ab1903) | Mouse | 1:10,000 | FA+PC | Rabbit anti-mouse biotinylated |
| TDP-43 | Abnova  (H00023435-M01) | Mouse | 1:800 | PC | Rabbit anti-mouse biotinylated |

Abbreviations: FA, formic acid; PC, pressure cooker; p-tau, phosphorylated tau

**Table S3**. Microglial protein location of expression and function [^21,22,29,30^](https://sciwheel.com/work/citation?ids=1246636,4934618,4618087,925564&pre=&pre=&pre=&pre=&suf=&suf=&suf=&suf=&sa=0,0,0,0&dbf=0&dbf=0&dbf=0&dbf=0).

| **Microglial protein** | **Predominant expression location** | **Microglial function** | **Microglial activation state** |
| --- | --- | --- | --- |
| **Iba1** | Cytoplasm | Cytoskeletal support and motility | Resting homeostatic and activated |
| **CD68** | Lysosome | Phagocytosis | Activated |
| **CR3-43** | Cell surface | Antigen presentation | Activated |


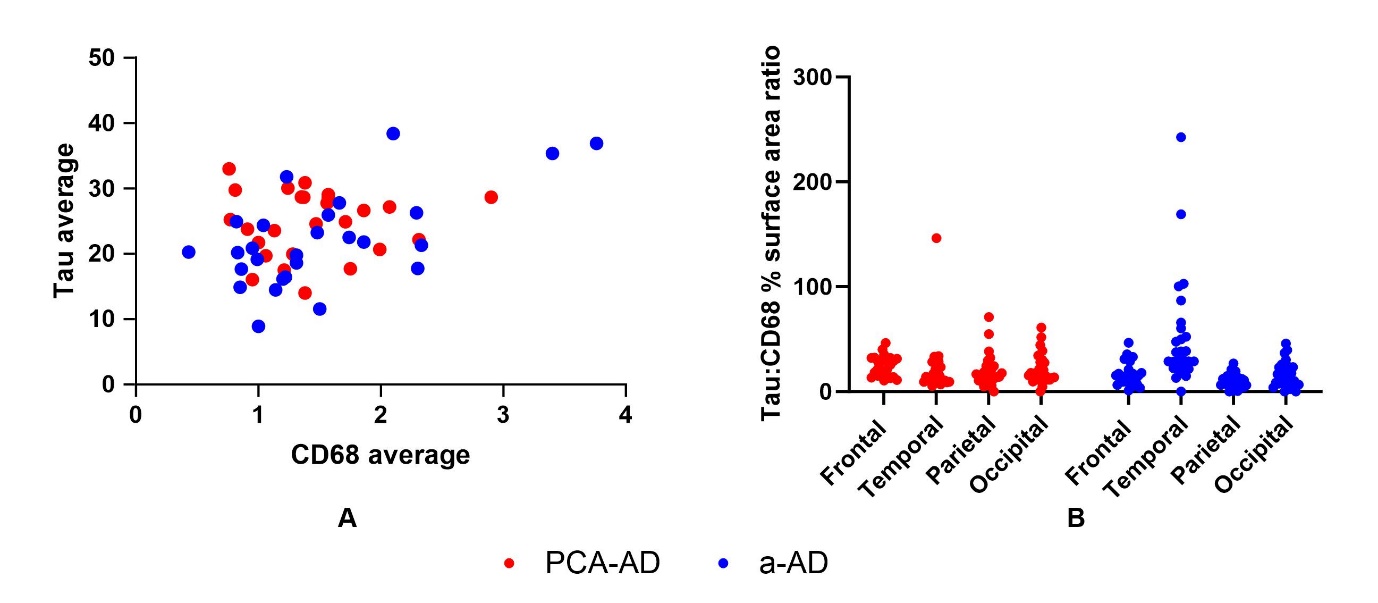


**Figure S1.** Relationship between tau and CD68.

(A) correlation between CD68 and tau showing positive association between CD68 and tau load globally (CD68 and tau load has been averaged across all four brain regions for PCA-AD and a-AD). (B) Tau to CD68 % surface area ratio in PCA-AD and a-AD cases.

**Table S4**. Estimated group differences [95% CIs] in Aβ, tau and CD68 markers (%area) at each region. Positive values indicate higher load in PCA-AD relative to a-AD.

|  | **Frontal** | **Temporal** | **Parietal** | **Occipital** |
| --- | --- | --- | --- | --- |
| **Aβ** | 1.16%  [-0.98, 3.30] | 0.77%  [-1.37, 2.91] | 2.51%  [0.37, 4.66] | 0.68%  [-1.49, 2.86] |
| **Tau** | 4.09%  [-0.54, 8.72] | -1.49%  [-6.15, 3.18] | 5.15%  [0.45, 9.85] | 1.98%  [-2.76, 6.71] |
| **CD68** | -0.49%  [-0.97, -0.01] | 0.78%  [0.31, 1.26] | -0.43%  [-0.90, 0.04] | -0.22%  [-0.69, 0.26] |

**PCA-plus and pathological observations**

Averaging across regions, there was no evidence that overall Aβ or tau load differed between PCA-AD and a-AD groups whether including or excluding the PCA-AD case with PCA-plus clinical features.

There was evidence for higher parietal tau load in the PCA-AD compared to a-AD group when including or excluding the PCA-plus case (estimated mean increase in tau surface area with: 5.2% (95% CI [0.5, 9.9]) without: 5.2% (95% CI [0.5, 9.8])). However, evidence for higher parietal Aβ load in the PCA-AD compared to a-AD group was weaker and formally non-statistically significant when excluding this case (estimated mean increase in Aβ surface area with: 2.5% (95% CI [0.4, 4.7]; without: 1.8% (95% CI [-0.1, 3.8])).

***ApoE* and pathological observations**

Inclusion of *ApoE4* carrier status did not improve model fit for regional Aβ/tau/microglial load or CAA severity scores using the LRT and was therefore not further analysed. Observed data for tau load however showed a tendency towards higher tau load in the parietal region of the *APoE4* carrier PCA group in particular, but this observation was not statistically significant.

Using Fisher’s exact test, there were no significant differences in α-syn presence/absence in PCA (p=0.375) or TAD (p=0.398) based on *ApoE4* carrier status. Similarly, there was no significant difference in TDP-43 presence/absence p=1.000 in PCA (p=1.000) or TAD (p=0.706) based on *ApoE4* carrier status.
